# The Interaction between Genetic Polymorphisms in *FTO* and *TCF7L2* Genes and Dietary Intake with Regard to Body Mass and Composition: An Exploratory Study

**DOI:** 10.3390/jpm9010011

**Published:** 2019-02-05

**Authors:** Lara Nasreddine, Reem Akika, Aurelie Mailhac, Hani Tamim, Nathalie Khoueiry Zgheib

**Affiliations:** 1 Department of Nutrition & Food Sciences, Faculty of Agriculture and Food Sciences, American University of Beirut, Beirut, PO Box 11-0236, Riad El-Solh 1107 2020, Lebanon; ln10@aub.edu.lb; 2 Department of Pharmacology and Toxicology, Faculty of Medicine, American University of Beirut, Beirut, PO Box 11-0236, Riad El-Solh 1107 2020, Lebanon; ra223@aub.edu.lb; 3 Clinical Research Institute, Faculty of Medicine, American University of Beirut, Beirut, PO Box 11-0236, Riad El-Solh 1107 2020, Lebanon; am138@aub.edu.lb; 4 Department of Internal Medicine, Faculty of Medicine, American University of Beirut, PO Box 11-0236, Riad El-Solh 1107 2020, Beirut, Lebanon

**Keywords:** nutrigenetics, *FTO*, *TCF7L2*, body mass, body mass composition

## Abstract

In contrast to the large number of genetic studies on obesity, there has been significantly less nutrigenetics investigation of the interaction between diet and single nucleotide polymorphisms (SNPs) in obesity, especially within Eastern Mediterranean populations. The aim of this study was to evaluate the potential interactions between three candidate SNPs, namely, *rs1558902* and *rs9939609* in the *fat mass and obesity* (*FTO)* gene and the *rs7903146* variant of the *Transcription factor 7 like 2* (*TCF7L2)* gene, and macronutrient intake with regard to obesity, body fat, and muscle composition. Three hundred and eight healthy Lebanese adults were included in this study. Data collection included a questionnaire for demographics and lifestyle in addition to a detailed dietary assessment using a culture-specific 80-item semi-quantitative food frequency questionnaire. This was coupled with anthropometric measurements and peripheral blood withdrawal for DNA and genotyping using Taqman allele discrimination assays. The two *FTO* candidate SNPs were not associated with risk of obesity in this population sample, yet there was a trend, though not a significant one, towards lower muscle mass among carriers of the risk allele of either *FTO* SNPs. To our knowledge, these results have not been previously reported. As for the *TCF7L2* *rs7903146* variant, results were congruent with the literature, given that individuals who were homozygous for the risk allele had significantly higher body mass index (BMI) and body fat despite lower intakes of saturated fat. Similar interactions, though not significant, were shown with muscle mass, whereby individuals who were homozygous for the risk allele had lower muscle mass with higher intakes of saturated fat, a result that, to our knowledge, has not been previously reported.

## 1. Introduction

Obesity is increasingly recognized as a public health concern with its prevalence reaching alarming levels in several parts of the world, plaguing both high- and low-income countries and jeopardizing their ability to cope with the increasing cost of treating obesity-associated diseases [1,2,3,4,5,6]. In Lebanon, a small Arab country in the Eastern Mediterranean basin, data stemming from national cross-sectional surveys has indicated a significant increasing trend in the prevalence of obesity across all age groups [7,8]. Increases in adult mean body mass index (BMI) have been estimated at 1.4 kg/m^2^ for Lebanese women and 1.8 kg/m^2^ for men over the past decade. This exceeds BMI increases reported from high-income countries such as the USA (1.1–1.2 kg/m^2^ per decade) [9] and exceeds a recently reported worldwide estimate (0.4–0.5 kg/m^2^ per decade). These findings carry important public health implications given that the association between body mass index (BMI) and chronic diseases is continuous and entails a dose-response relationship [3,9].

The causes of weight gain and obesity are multifactorial, and efforts to identify these causes are crucial for the development of effective preventive strategies. In addition to high dietary energy intake and low energy expenditure, obesity is influenced by factors such as genetic predisposition. Although hereditability seems substantial, with at least a 40% contribution based on twin and family studies, genetic mechanisms predisposing people to obesity are still not very well understood and are at many times contradictory [10,11]. Recent studies have shown that genetic variants interact with environmental factors such as food intake and hence affect obesity. The evaluation of this interaction is termed nutrigenetics, the aim of which is to better understand an individual phenotype by genetic predisposition. It also applies to the concept of “personalized nutrition”, which aims to prevent the onset and development of chronic diseases by targeting dietary recommendations to an individual’s genetic profile [10,11].

In contrast to the large number of genetic studies on obesity [12] there has been significantly less nutrigenetics research on the interaction between diet and single nucleotide polymorphisms (SNPs) with regard to obesity. The highest evidence by far is for the two *rs1558902* and *rs9939609* variants in the *fat mass and obesity* (*FTO*) associated gene, with the *A* allele being consistently linked to increasing BMI [13] as well as being more prone to weight loss through diet or lifestyle interventions in comparison to non-carriers [14]. This risk allele has also been shown to modify associations/responses to protein, fat, and exercise for weight-related phenotypes. For example, homozygous *AA* genotypes have been seen to produce greater reductions in weight, increases in muscle mass and changes in fat distribution in response to a two-year high protein diet, with opposite effects having been observed in those who consumed low protein diets [15]. As for gene interaction with fat intake, *AA* individuals are also prone to significantly enhanced weight loss in response to consuming a diet that is low in saturated fat and high in polyunsaturated fat [16], and to more weight gain with high fat and carbohydrate diets [17]. Interestingly, participants with the *AA* genotype have an enhanced weight loss response with higher physical activity when compared to *TT* [18]. It is important to note that these SNPs are in high-linkage disequilibrium (LD) in Europeans, though it is not known if this is the case for the Eastern Mediterranean population. An additionally interesting fact is the implication of variants in the *Transcription factor 7 like 2* (*TCF7L2*) gene with fat interactions. For example, in a 10-week randomized hypoenergetic diet, *TCF7L2 rs7903146* homozygous *TT* participants were seen to significantly lose 44% more weight in response to a moderate- to low-fat diet [19]. A two-year weight loss intervention has also showed congruent results [20].

To our knowledge, there have been no diet-gene interaction evaluations for the Eastern Mediterranean population except for a couple of studies on dietary interaction with candidate SNPs in *Melanocortin 4 Receptor* (*MC4R*) and the *Apolipoprotein* gene family in Iranian populations [21,22,23]. Therefore, the aims of this nutrigenetics study were to evaluate the potential interaction between three candidate SNPs, *rs1558902* and *rs9939609* in the *FTO* gene and the *rs7903146* variant of the *TCF7L2* gene, and macronutrient intake with regard to obesity, body fat and muscle composition.

## 2. Materials and Methods

### 2.1. Study Participants and Data Collection

This cross-sectional study builds on a previously recruited and described community cohort of unrelated Lebanese individuals [24]. All subjects gave their informed consent for inclusion before they participated in the study. The study was conducted in accordance with the Declaration of Helsinki and the protocol was approved by the Ethics Committee of the American University of Beirut (Pharmaco.NZ.23).

After signing their informed consent, 501 Lebanese subjects older than 18 years old were recruited from the Greater Beirut area between February and June 2014. A multi-component questionnaire was administered in an interview settin, to obtain information on demographic and socioeconomic characteristics, lifestyle habits, physical activity using the short version of the International Physical Activity Questionnaire (IPAC) [25], and medical history. Fasting blood samples were obtained for glucose, hemoglobin A1C (HbA1C), and lipid profile measurements. Sitting blood pressure was obtained twice at 10-min intervals using a digital sphygmanometer.

For the present study, participants who reported the diagnosis of a chronic disease or metabolic abnormality that could have affected their food consumption habits were excluded. The selection of subjects from the original survey (*n* = 501) was therefore performed based on the following criteria: Firstly, having complete anthropometric and dietary data, and secondly, having no known diagnosis of hypertension, dyslipidemia, diabetes, or hyperglycemia. The final sample size was 308.

### 2.2. Anthropometric Measurements

Anthropometric measurements were obtained using standardized protocols [26] and calibrated equipment. Height and weight measurements were collected using a portable stadiometer (Holtain, Crymych, UK) and a Seca calibrated electronic weighing scale (Hamburg, Germany), respectively. The measurements were taken twice and the average of the two values adopted. Body mass index was calculated as the ratio of weight (kilograms) to the square of height (meters). A tetrapolar single frequency (330 µA at 100 kHz) electrical bioimpedance analyzer (Inbody Body Composition Analyzer, Inbody 230, InBody Co., Ltd., Seoul, Korea) was used to measure body composition.

### 2.3. Dietary Data

Dietary intake was assessed using a culture-specific 80-item semi-quantitative food frequency questionnaire (FFQ) [27]. Participants were asked to specify the frequency of consumption as either per day, per week, per month, per year or never. Participants had the choice to report their intake either in terms of reference portion size or in grams. The reference portions of the two-dimensional food portion visual (Millen and Morgan, Nutrition Consulting Enterprises, Framingham, MA, USA) [28] were used, and a reference portion, representing one standard serving expressed in household measures, was defined for each food item. Common household measures used were measuring cups and spoons, in addition to real portion-size photographs. The reported frequency of each food item and beverage was then converted to a daily portion intake. The daily energy and macronutrient consumption of participants was computed using the food composition database of Nutritionist Pro™ software (Axxya Systems LLC, Stafford, TX, USA) and a food composition table of Middle-Eastern foods for local and traditional foods [29].

### 2.4. Genotyping

DNA was isolated from 300 µL peripheral whole blood using the Qiagen Flexigene^®^ DNA kit (Qiagen, Hilden, Germany, cat # 51204) and stored at −20 °C until analysis. Genotyping for the three candidate SNPs was performed using Taqman allele discrimination assays on the CFX384 Touch real-time PCR detection system from BioRad (Hercules, CA, USA). The reaction mix contained 2× universal PCR Master Mix No AmpErase UNG (ThermoFisher Scientific, Applied Biosystems, Waltham, MA, USA) and 40× Taqman SNP genotyping assay (ThermoFisher Scientific, Waltham, MA, USA) diluted to 1×, DNA, and Nuclease-free water to make up a total volume of 10 µL/well. The PCR protocol was as described by the manufacturer: 95 °C for 10 min, then 40 cycles of 92 °C for 15 s followed by 60 °C for 1 min. Ten percent of the samples were run in duplicate and showed 100% reproducibility.

### 2.5. Statistical Analyses

Data were analyzed using Statistical Package for Social Sciences (SPSS) version 24.0 for Windows (Chicago, IL, USA). Since there were three groups of analyses (BMI, body fat, and muscle mass), Bonferroni’s correction was used for the *p*-value considered in this study and the statistical significance level was set at 0.016 (being the ratio of 0.05 over three). No power analysis was performed because the study aims were multifactorial and included interactions with a number of dietary factors on three outcomes with potential confounders. Moreover, there is little nutrigenetics literature on body fat and muscle mass, in addition to the fact that this study builds on an already available sample. Thus, this work is considered exploratory and hypothesis generating.

Allele frequencies were calculated and tested for Hardy Weinberg Equilibrium (HWE) using the chi square test. A Kappa statistic for agreement was calculated for the two *FTO* genotypes for assessment of LD. Note that all of the participants were Caucasian Arabs.

Data were described in terms of frequencies and percentages for categorical variables, and mean ± standard deviation (SD) for continuous ones. Means with associated 95% confidence intervals (CI) of continuous variables were also estimated where appropriate. Associations between genotypes and participant characteristics, anthropometric measurements, lifestyle factors, and diet were performed using chi square or one-way analysis of variance (ANOVA) as applicable.

Dietary intakes of macronutrients, expressed as percent energy intakes, were categorized into tertiles based on statistical grounds. A within-tertile trend analysis was performed using general linear models. The results were adjusted for potentially confounding factors, namely, age, sex and physical activity, as applicable. To account for any effect modification of dietary intake on the associations between the genotypes and each of the three outcomes, a multivariate analysis was carried out while including an interaction term of the genotypes and the different dietary intake tertiles. The same approach was carried out for the effect modification of physical activity on the various associations.

## 3. Results

### 3.1. Genotype Frequencies

The minor allele frequencies (MAFs) of the three SNPs were: *FTO rs1558902 A* allele (46%), *FTO rs9939609 A* allele (45%), and *TCF7L2 rs7903146 T* allele (35%). These were all in HWE and the frequencies were similar to those for reported MAFs [30]. As expected, the two *FTO* SNPs were in Linkage Disequilibrium (LD) (Kappa (95% CI) = 0.83(0.78–0.89)), and therefore, data for *FTO rs9939609* are shown in supplementary tables only. 

### 3.2. Associations of Baseline Characteristics, Lifestyle, and Dietary Habits with Genotypes

Table 1 shows the characteristics, lifestyle, and dietary habits of the study sample and the association of these characteristics with *FTO rs1558902* and *TCF7L2 rs7903146*. The majority of the included participants were female (62.7%); they were relatively young with a mean ± SD age of 39.79 ± 14.00 years. The majority performed some type of exercise (85.7%). There was a relatively high proportion of current smokers (43.8% for cigarette and 32.8% for narghileh), and approximately one fifth of the study population currently consumed alcohol (21.4%).

Mean daily dietary energy intake was estimated at 3600.55 ± 2029.45 Kcal/day with carbohydrates making up the highest contribution (50.07%), followed by fat (39.00%), and protein (12.95%). Sugar was estimated to contribute 14.8% of energy intake, while the percent contributions of the various types of fat were as follows: 10.38% for saturated fat, 13.8% for monounsaturated fat (MUFA), and 10% for polyunsaturated fat (PUFA).

None of these factors differed between the genotypes except for age, which differed in terms of the *FTO* genotypes only (Table 1 and Table S1).

### 3.3. Associations of BMI and Body Fat and Muscle with Genotypes

As shown in Table 1, the average BMI for the study population was estimated at 27.78 ± 5.62 kg/m^2^. There was a high prevalence of being overweight or obese in the study sample, with 66.2% being overweight (BMI > 25 kg/m^2^) and 31.2% being obese (>30 kg/m^2^). Mean body fat was estimated at 26.24 ± 11.42 kg and muscle mass at 26.43 ± 6.37 kg. No significant differences were noted among the different genotypes (Table 1 and Table S1).

### 3.4. Interactions between Diet and Genotypes with BMI, Body Fat, and Muscle Mass

**Figure 1:**
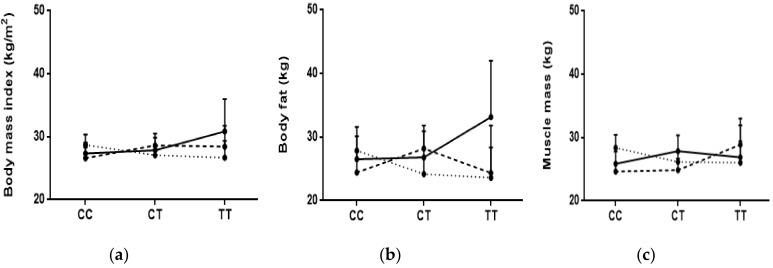
Interactions between tertiles of daily % energy saturated fat intake and *TCF7L2* (*rs7903146*) genotypes with (**a**) BMI (kg/m^2^), (**b**) body fat (kg) and (**c**) muscle mass (kg). Dots represent mean values and bars represent the upper 95% confidence interval: ____ <8.595 %; **----** 8.595–11.034%; …… >11.034%.

For BMI, and as shown in Table 2 and Table 3 and Table S2, there were trends with the *TCF7L2* SNP whereby subjects who were homozygous for the risk allele (*TT*) and categorized in the second tertile of % of energy fat intake were significantly heavier (mean (95%CI): 31.03 (26.64–35.43)) even after adjustment for age, sex, and physical activity. More importantly, there was a significant interaction between % of energy saturated fat and the *TCF7L2* genotypes, in which it appears that those homozygous for the risk allele had a higher BMI despite lower intakes of saturated fat (tertiles 1 and 2) (Table 3 and Figure 1a). This significance appeared after adjustment for age, sex, and physical activity. No significant trends or interactions were revealed with both *FTO* SNPs.

Concerning body fat, and as shown in Tables S3–S5, there was also a trend, though non-significant, with the *TCF7L2* SNP, whereby subjects who were homozygous for the risk allele (*TT*) and categorized in the second tertile of % of energy fat intake were heavier (mean (95% CI): 31.34 (22.69–39.98)) (*p*-values between 0.05 and 0.016 after adjustment). More importantly, there was, again and similarly to BMI, a significant interaction between % of energy saturated fat and the *TCF7L2* genotypes whereby those homozygous for the risk allele had higher body fat despite lower intakes of saturated fat (tertile 1) (Table S4 and Figure 1b). This significance appeared after adjustment for age, sex, and physical activity. No significant trends or interactions were revealed with both *FTO* SNPs.

With regard to muscle mass (Tables S6–S8), there was a non-significant trend (*p* = 0.04) with the *FTO rs1558902* SNP, whereby participants who were homozygous for the risk allele (*AA*) had lower muscle mass when compared to those who were heterozygous or homozygous for the non-risk allele. This was true for the whole cohort and after categorization of dietary intake into tertiles (*p*-values between 0.05 and 0.016) (Table S6). Similar results appeared with *FTO rs9939609* (Table S8). There was also a trend with the *TCF7L2* SNP, though not significant, whereby subjects who were homozygous for the risk allele (*TT*) and categorized in the second tertile of % of energy polyunsaturated fat intake had higher muscle mass (mean (95%CI): 30.77 (26.34–35.20)) after adjustment (*p*-values between 0.05 and 0.016) (Table S7). Interestingly, there was an interaction, though not significant, between % of energy saturated fat and the *TCF7L2* genotypes, whereby individuals who were homozygous for the risk allele had lower muscle mass with high intakes of saturated fat (tertile 3) (Table S7 and Figure 1c). No significant interactions were revealed with both *FTO* SNPs.

Of note is that no significant interactions were revealed between physical activity and the three SNPs concerning either BMI or body composition (Table S9).

## 4. Discussion

This is the first nutrigenetics study performed on a Lebanese population. We have shown that although the two *FTO* candidate SNPs are not associated with risk of obesity in this population sample, there was a trend, though not significant, towards lower muscle mass among carriers of the risk allele of both *FTO SNPs.* To our knowledge, these results have not been reported previously. As for the *TCF7L2 rs7903146* variant, we have shown results that are congruent with the literature [19,20] whereby individuals who were homozygous for the risk allele had significantly higher BMI and body fat despite lower intakes of saturated fat. Similar interactions, though not significant, were shown with muscle mass whereby those homozygous for the risk allele had lower muscle mass with high intakes of saturated fat, a result that to our knowledge, has not been previously reported. Note that none of the three SNPs were associated with differences in macronutrient intake, a finding that was established in a recent genome-wide meta-analysis [31].

In addition to *MC4R*, loci in the *FTO* gene have been consistently linked with the risk of obesity [32,33,34,35,36,37] with *FTO* being highly expressed in the hypothalamus and playing a role in the ghrelin, leptin, and melanocortin pathways [38,39,40]. The most compelling data has been, however, mainly compiled from Europeans and individuals of European descent [32,33,34,35,36]. Interestingly, in a meta-analysis of genome-wide association studies of those from diverse ancestries (five Caucasians, one Chinese, one African-American, and one Hispanic), it was shown that the significant *FTO* SNPs that were revealed in Caucasians had no or weak evidence of association in non-Caucasian groups [41]. These findings and the interesting result of this study concerning a potentially lower muscle mass among the *FTO* risk allele carriers reinforce the potential for uniqueness in differences in genetic variations in various populations and their differential role in obesity risk, although the current study may be insufficiently able to detect a significant association between *FTO* SNPs and body mass and composition. As for the potential interaction between *FTO* SNPs and diet with regard to body mass and composition, a recent systematic review and meta-analysis of 14 studies with various intervention types and durations has shown that individuals who carry the *FTO T* risk allele may benefit more from diet and lifestyle interventions when compared to non-carriers. Nevertheless, it is important to note that the benefit is relatively small (0.18 to 0.20 kg additional weight loss). In addition, a meta-analysis of only randomized controlled trials (*n* = 8, some of which were included in the aforementioned analysis) has shown that the *FTO risk* allele was not associated with any differential change in adiposity after dietary weight loss interventions [42]. It is possible that the lack of interactions with the two *FTO* variants in the current study is due to the study design being cross-sectional with a relatively small sample size when compared to, for example, Sonestedt and colleagues’ [17] evaluation of close to 5000 subjects. An additional compelling explanation could be interethnic differences as recently suggested by Merritt et al. [43] who showed that protein intake modifies the effect of the *FTO rs1558902* risk allele on body weight in East Asians but not in Caucasians or South Asians.

The *TCF7L2* gene, which is known to play an important role in the transcription of the proglucagon gene and hence the synthesis of glucagon-like-peptide 1 (GLP-1), which is implicated in insulin regulation, appetite and food intake [44,45], has been studied much less in the context of obesity and more in diabetes and metabolic syndrome [46,47]. To our knowledge, only a few studies have evaluated the potential of macronutrient-*TCF7L2* gene interaction with regard to obesity and weight loss [19,20,48,49]. Two of these have shown that individuals who are homozygous for the *TCF7L2 rs7903146* T-risk allele are more sensitive to low fat weight-loss diets [19,20] and one has revealed that *T* risk allele carriers are more prone to weight loss when they consume a Mediterranean diet that is rich in sources of unsaturated fat [49]. Interestingly, no significant differences in baseline measures of body mass and composition by genotype groups were observed. Results of the current study, though cross sectional by design, concur with these findings and suggest that the *T*-allele confers a higher risk for obesity and adiposity in spite of lower saturated fat intakes. Of note is that it is unclear to us why only those who were categorized in the second tertile of percent energy from total fat and were homozygous for the risk allele (*TT*) had significantly higher BMI values. This is a finding that necessitates further investigation and may have appeared in the two other tertiles with a larger sample size. More importantly, this study is the first to show a potential link between lower muscle mass in individuals who are homozygous for the *T*-risk allele and who consume higher levels of saturated fat. It is unclear to us why subjects who were homozygous for the risk allele (*TT*) and were categorized in the second tertile of percent energy from polyunsaturated fat had significantly higher muscle mass. Increasing evidence, however, points towards differential effects of various types of fatty acids on metabolic, regulatory, and biological processes, with the concept of fatty acid sensing gaining more recognition [50]. It has in fact been suggested that the type of dietary fat may be an important determinant of body composition and lean body mass [51]. One supplementation trial conducted among postmenopausal women has shown that PUFAs (safflower oil) increase lean tissue and reduced trunk fat [52]. Similarly, a randomized trial conducted among normal weight adults has shown that, in comparison with saturated fat, PUFAs (sunflower oil) promote lean tissue accretion, with the increase in lean body mass being nearly three times greater in comparison with saturated fat feeding [51]. In our study, the intake of saturated fat was estimated at around 10.4% of energy intake, with 56% of the study subjects exceeding the upper limit specified by the World Health Organization (WHO) (i.e., 10% of energy intake) [53]. This high intake of saturated fat may be of concern, particularly in light of its potential interaction with the *T*-risk allele. The consumption of saturated fat has been on the rise in Lebanon as the nutrition transition unfolds [8]. The traditional Lebanese diet, which is rich in fruits, vegetables, pulses, and olive oil, and which is described as a variant of the Mediterranean diet [54], is in fact gradually eroding and shifting towards a Westernized dietary pattern which is characterized by high intakes of animal products and fast food, with significantly higher levels of saturated fat [27].

This study has several strengths. It was conducted on a random sample of the adult population living in Greater Beirut and followed a well-planned design, protocol, and methodology. Rather than estimating total fat alone, the dietary assessment performed in our study allowed us to differentiate between the intakes of various types of fatty acids and to contribute to the growing body of evidence on the potential differential effects of these fatty acids. In addition, we did not rely on BMI as the sole indicator of adiposity but rather performed a comprehensive body composition assessment where both components of fat mass and muscle mass were evaluated.

The results of this study should, however, be considered in light of the following limitations, on top of the relatively small sample size. First, the dietary assessment performed in this study was based on an FFQ, which may be limited by measurement errors, reliance on memory, and the number of food items included in the food list. FFQs may also overestimate energy intake, which, coupled with the high prevalence of being overweight and obesity in our sample, may explain the observed high energy intake in the study population [55]. Despite its potential limitations, the FFQ method has been reported as one of the most suitable dietary assessment tools in large epidemiological studies since it provides information on a subject’s habitual diet over long periods of time [56]. It is worth noting that although the FFQ used in the present study was not validated in the study population, it was previously adopted for the assessment of dietary intakes and their association with obesity and the metabolic syndrome in Lebanese adults, yielding plausible findings [27,57,58,59]. In addition, the FFQ was administered by trained nutritionists rather than being self-administered. The self-administration approach has several disadvantages, as self-administration of the FFQ necessitates a literate population, and could result in inconsistent interpretations of the food list and be associated with lower response and completion rates, all of which may jeopardize the validity of the data [56]. However, as is generally observed in most questionnaire-based surveys, the interview approach may engender a social desirability bias, whereby participating subjects may answer in a manner that they perceive as acceptable or favorable to the interviewer [60]. In the present study, the field workers who performed data collection received thorough training to decrease judgmental verbal and non-verbal communication in order to reduce social desirability bias.

## 5. Conclusions

In conclusion, the current results add to the growing literature on gene-nutrient interaction with obesity [10,11]. Although variants in the *FTO* gene do not appear to interact with various macronutrients with regard to body mass and composition, altering dietary fat composition with respect to the *TCF7L2 rs7903146* variant may differentially affect body parameters. These results should, however, be confirmed with a larger population sample and preferably a prospective interventional cohort design should they be translated into a personalized nutrition practice.

## Figures and Tables

**Table 1:** Associations of baseline characteristics, lifestyle, and dietary habits for those with *FTO (rs9939609)* and *TCF7L2 (rs7903146)* genotypes.

		*FTO (rs1558902)*				*TCF7L2 (rs7903146)*			
	Among All	*TT*	*AT*	*AA*	*p*	CC	CT	TT	*p*
	*N* = 308	*N* = 85	*N* = 165	*N* = 58		*N* = 134	*N* = 130	*N* = 43	
***Characteristics and lifestyle factors***									
**Gender, Female**	193 (62.7)	50 (58.8)	103 (62.4)	40 (69.0)	0.47	86 (35.8)	82 (63.1)	25 (58.1)	0.77
**Age (years)**	39.79 ± 14.00	38.46 ± 13.59	41.85 ± 14.23	35.89 ± 13.04	**0.011**	38.80 ± 13.66	40.61 ± 14.39	40.70 ± 14.02	0.53
**Crowding index**	1.55 ± 0.85	1.55 ± 0.89	1.56 ± 0.87	1.49 ± 0.74	0.86	1.54 ± 0.82	1.57 ± 0.83	1.52 ± 1.04	0.93
**Levels of physical activity**									
Low	137 (44.5)	35 (41.2)	72 (43.6)	30 (51.7)	0.71	51 (38.1)	68 (52.3)	18 (41.9)	0.20
Moderate	99 (32.1)	27 (31.8)	55 (33.3)	17 (29.3)		50 (37.3)	34 (26.2)	14 (32.6)	
High	72 (23.4)	23 (27.1)	38 (23.0)	11 (19.0)		33 (24.6)	28 (21.5)	11 (25.6)	
**Physical activity**									
None	44 (14.3)	14 (16.5)	20 (12.1)	10 (17.2)	0.50	17 (12.7)	18 (13.9)	9 (20.9)	0.40
Any	264 (85.7)	71 (83.5)	145 (87.9)	48 (82.8)		117 (87.3)	112 (86.2)	34 (79.1)	
**Cigarette smoker**									
Never	148 (48.1)	43 (50.6)	73 (44.2)	32 (55.2)	0.23	65 (48.5)	59 (45.4)	24 (55.8)	0.71
Current	135 (43.8)	32 (37.7)	79 (47.9)	24 (41.4)		60 (44.8)	58 (44.6)	16 (37.2)	
Past	25 (8.1)	10 (11.8)	13 (7.9)	2 (3.5)		9 (6.7)	13 (10.0)	3 (7.0)	
**Narghileh smoker**									
Never	177 (57.5)	43 (50.6)	103 (62.4)	31 (53.5)	0.28	81 (60.5)	75 (57.7)	20 (46.5)	0.62
Current	101 (32.8)	32 (37.7)	46 (27.9)	23 (39.7)		41 (30.6)	42 (32.3)	18 (41.9)	
Past	30 (9.7)	10 (11.8)	16 (9.7)	4 (6.9)		12 (9.0)	13 (10.0)	5 (11.6)	
**Alcohol drinker**									
Never	223 (72.4)	60 (70.6)	119 (72.1)	44 (75.9)	0.38	100 (74.6)	99 (76.2)	24 (55.8)	0.08
Current	66 (21.4)	20 (23.5)	38 (23.0)	8 (13.8)		28 (20.9)	22 (16.9)	15 (34.8)	
Past	19 (6.2)	5 (5.9)	8 (4.9)	6 (10.3)		6 (4.5)	9 (6.9)	4 (9.3)	
***Body mass and composition***									
**Body mass index (BMI) (kg/m²)**	27.78 ± 5.62	28.49 ± 5.70	27.35 ± 5.44	27.96 ± 6.00	0.31	27.57 ± 5.65	27.79 ± 5.44	28.37 ± 6.20	0.72
**Body fat (kg)**	26.24 ± 11.42	27.48 ± 11.72	25.30 ± 11.09	27.12 ± 11.84	0.29	26.27 ± 11.73	26.15 ± 10.91	26.32 ± 12.30	0.99
**Muscle mass (kg)**	26.43 ± 6.37	27.41 ± 6.71	26.35 ± 6.19	25.21 ± 6.25	0.13	26.32 ± 6.14	26.17 ± 6.33	27.25 ± 7.09	0.62
***Energy and macronutrient intake***									
**Total energy (Kcal/day)**	3600.55 ± 2029.45	3737.20 ± 1973.73	3491.42 ± 2048.33	3710.71 ± 2072.60	0.60	3728.28 ± 2359.00	3467.58 ± 1782.08	3632.36 ± 1608.44	0.58
**Carbohydrates (g/day)**	441.61 ± 254.55	475.47 ± 271.82	427.01 ± 253.47	437.90 ± 230.63	0.41	468.61 ± 317.18	416.61 ± 195.45	435.43 ± 182.07	0.25
**Percent Kcal from carbohydrates (%)**	50.07 ± 8.54	51.24 ± 8.85	49.89 ± 7.79	48.85 ± 9.95	0.24	50.98 ± 8.62	49.27 ± 8.40	49.47 ± 8.63	0.24
**Proteins (g/day)**	116.26 ± 77.07	113.44 ± 61.23	113.44 ± 68.02	129.24 ± 113.76	0.36	115.59 ± 75.04	117.14 ± 84.40	116.65 ± 60.46	0.99
**Percent Kcal from proteins (%)**	12.95 ± 3.66	12.32 ± 2.72	13.15 ± 3.44	13.32 ± 5.13	0.16	12.61 ± 2.85	13.41 ± 4.58	12.70 ± 2.58	0.18
**Sugar (g/day)**	132.55 ± 117.60	146.90 ± 173.91	125.58 ± 91.12	131.37 ± 74.63	0.40	147.88 ± 156.56	120.28 ± 73.09	123.54 ± 75.09	0.14
**Percent Kcal from sugar (%)**	14.79 ± 6.47	14.82 ± 7.30	14.62 ± 5.88	15.22 ± 6.90	0.83	15.59 ± 7.08	14.37 ± 6.03	13.70 ± 5.65	0.15
**Total fat (g/day)**	150.12 ± 90.40	147.92 ± 76.68	147.09 ± 91.08	162.00 ± 106.31	0.54	151.61 ± 97.79	148.71 ± 86.89	151.30 ± 78.34	0.96
**Percent Kcal from total fat (%)**	39.00 ± 7.89	37.65 ± 7.52	39.30 ± 7.43	40.14 ± 9.43	0.14	38.39 ± 7.90	39.76 ± 7.61	38.75 ± 8.72	0.36
**Saturated fat (g/day)**	42.97 ± 29.24	42.62 ± 24.57	42.07 ± 30.95	46.04 ± 30.78	0.67	44.06 ± 33.05	42.34 ± 26.84	42.03 ± 23.65	0.87
**Percent Kcal from saturated fat (%)**	10.38 ± 2.76	10.17 ± 2.66	10.39 ± 2.80	10.65 ± 2.84	0.60	10.22 ± 2.65	10.65 ± 2.98	10.11 ± 2.40	0.34
**Monounsaturated fat (MUFA) (g/day)**	55.33 ± 34.48	54.70 ± 29.58	54.35 ± 34.55	59.04 ± 40.76	0.66	55.43 ± 35.99	54.73 ± 33.52	57.29 ± 33.46	0.92
**Percent kcal from MUFA (%)**	13.82 ± 4.03	13.37 ± 3.78	13.98 ± 4.06	14.02 ± 4.32	0.49	13.47 ± 3.85	14.07 ± 3.81	14.17 ± 5.14	0.40
**Polyunsaturated fat (PUFA) (g/day)**	39.36 ± 25.06	38.07 ± 20.26	38.55 ± 24.34	43.52 ± 32.42	0.37	39.42 ± 24.49	39.36 ± 25.64	39.55 ± 25.80	1.00
**Percent kcal from PUFA (%)**	10.00 ± 3.76	9.40 ± 3.17	10.13 ± 3.78	10.54 ± 4.41	0.17	9.84 ± 3.60	10.18 ± 3.64	10.00 ± 4.63	0.77
**Cholesterol (mg/day)**	382.03 ± 373.27	398.50 ± 460.86	358.75 ± 295.23	424.11 ± 427.62	0.46	388.62 ± 403.55	382.10 ± 365.80	352.42 ± 295.41	0.86

Data are presented as mean ± standard deviation of the mean (SD) or *N* (%). *p* values: chi-square, *t*-test or one-way analysis of variance (ANOVA) as appropriate; *p*-values that are statistically significant are in bold.

**Table 2:** Interaction between daily dietary intake and *FTO* (*rs1558902*) genotypes with BMI (kg/m^2^).

	No. of Subjects by Genotype (*TT*/*AT*/*AA*)	*TT* Mean (95% CI) (*N* = 85)	*AT* Mean (95% CI) (*N* = 165)	*AA* Mean (95% CI) (*N* = 58)	*p*-Trend ^1^	*p*-Trend ^2^	*p*-Trend ^3^	*p*-Interaction ^1^	*p*-Interaction ^2^	*p*-Interaction ^3^
**Among ALL**	85/165/58	28.49 (27.26–29.72)	27.35 (26.52–28.19)	27.96 (26.38–29.54)	0.58	0.68	0.53	-	-	-
**Carbohydrates (% of energy)**										
Tertile 1 (<47.317)	25/53/26	29.05 (26.88–31.22)	27.63 (26.24–29.02)	28.49 (25.93–31.04)	0.71	0.43	0.44	0.58	0.76	0.73
Tertile 2 (47.317–53.984)	29/64/15	27.33 (25.30–29.35)	27.03 (25.63–28.43)	27.25 (24.04–30.45)	0.96	0.70	0.76			
Tertile 3 (>53.984)	31/48/17	29.12 (26.78–31.45)	27.47 (25.81–29.13)	27.78 (24.71–30.84)	0.46	0.59	0.43			
**Fat (% of energy)**										
Tertile 1 (<35.131)	31/45/16	28.74 (26.31–31.17)	26.92 (25.13–28.71)	29.27 (25.79–32.76)	0.79	0.72	0.94	0.86	0.98	0.81
Tertile 2 (35.131–41.333)	27/67/17	27.59 (25.60–29.57)	28.08 (26.78–29.38)	24.87 (23.07–26.67)	0.08	0.24	0.21			
Tertile 3 (>41.333)	27/53/25	29.09 (26.99–31.18)	26.80 (25.39–28.21)	29.21 (26.57–31.86)	0.93	0.98	0.98			
**Protein (% of energy)**										
Tertile 1 (<11.700)	39/57/25	29.34 (27.93–30.75)	27.10 (25.82–28.39)	27.12 (24.53–29.70)	0.09	0.10	0.06	0.55	0.44	0.41
Tertile 2 (11.700–13.985)	27/54/15	26.10 (23.98–28.29)	26.78 (25.26–28.29)	29.21 (26.19–32.23)	0.08	0.06	0.06			
Tertile 3 (>13.985)	19/54/18	30.13 (26.47–33.78)	28.19 (26.59–29.80)	28.08 (24.99–31.17)	0.33	0.22	0.16			
**Saturated fat (% of energy)**										
Tertile 1 (<8.595)	26/42/17	28.11 (26.31–29.92)	27.73 (25.80–29.65)	28.65 (25.13–32.17)	0.77	0.47	0.52	0.35	0.29	0.33
Tertile 2 (8.595–11.034)	28/65/11	28.18 (25.70–30.65)	27.36 (25.95–28.78)	28.46 (24.10–32.82)	0.90	0.98	0.83			
Tertile 3 (>11.034)	31/58/30	29.07 (26.84–31.31)	27.07 (25.87–28.27)	27.38 (25.34–29.42)	0.21	0.20	0.19			
**MUFA (% of energy)**										
Tertile 1 (<11.890)	29/44/19	29.12 (26.60–31.65)	26.54 (25.02–28.07)	29.05 (26.16–31.94)	0.97	0.72	0.89	0.89	0.95	0.92
Tertile 2 (11.890–14.571)	27/65/18	27.87 (25.82–29.92)	28.05 (26.72–29.37)	26.36 (23.40–29.33)	0.36	0.54	0.63			
Tertile 3 (>14.571)	29/56/21	28.42 (26.42–30.42)	27.18 (25.62–28.75)	28.33 (25.59–31.08)	0.96	0.83	0.73			
**PUFA (% of energy)**										
Tertile 1 (<8.426)	36/51/21	28.81 (26.59–31.04)	27.32 (25.46–29.19)	27.62 (24.71–30.53)	0.51	0.84	0.66	0.34	0.49	0.41
Tertile 2 (8.426–10.930)	26/66/16	28.45 (26.25–30.66)	27.16 (26.01–28.01)	27.30 (24.88–29.72)	0.46	0.30	0.13			
Tertile 3 (>10.930)	23/48/21	28.01 (26.03–29.98)	27.64 (26.16–29.12)	28.80 (25.73–31.86)	0.63	0.64	0.66			

^1^ *p*-value crude; ^2^ *p*-value adjusted for age and sex; ^3^ *p*-value adjusted for age, sex, and physical activity.

**Table 3:** Interaction between daily dietary intake and *TCF7L2* (*rs7903146*) genotypes with BMI (kg/m^2^).

	No. of Subjects by Genotype (*CC*/*CT*/*TT*)	*CC* Mean (95% CI) (*N* = 134)	*CT* Mean (95% CI) (*N* = 130)	*TT* Mean (95% CI) (*N* = 43)	*p*-Trend ^1^	*p*-Trend ^2^	*p*-Trend ^3^	*p*-Interaction ^1^	*p*-Interaction ^2^	*p*-Interaction ^3^
**Among ALL**	134/130/43	27.57 (26.61–28.54)	27.79 (26.84–28.73)	28.37 (26.46–30.28)	0.42	0.47	0.48	-	-	-
**Carbohydrates (% of energy)**										
Tertile 1 (<47.317)	43/46/15	28.96 (27.29–30.63)	27.95 (26.44–29.46)	26.69 (23.18–30.20)	0.17	0.20	0.20	0.10	0.11	0.17
Tertile 2 (47.317–53.984)	44/49/15	26.07 (24.24–27.89)	27.62 (26.13–29.10)	28.74 (26.11–31.38)	0.11	0.15	0.12			
Tertile 3 (>53.984)	47/35/13	27.71 (26.16–29.27)	27.82 (25.64–30.00)	29.87 (25.39–34.36)	0.25	0.30	0.33			
**Fat (% of energy)**										
Tertile 1 (<35.131)	47/28/16	27.69 (25.80–29.58)	28.58 (25.80–31.35)	27.53 (25.10–29.96)	0.93	0.87	0.87	0.24	0.35	0.33
Tertile 2 (35.131–41.333)	46/51/14	26.10 (24.85–27.35)	27.72 (26.45–29.00)	31.03 (26.64–35.42)	**0.001**	**0.003**	**0.002**			
Tertile 3 (>41.333)	41/51/13	29.09 (27.26–30.93)	27.42 (25.94–28.90)	26.53 (23.06–30.01)	0.15	0.21	0.19			
**Protein (% of energy)**										
Tertile 1 (<11.700)	53/50/18	28.01 (26.53–29.49)	27.64 (26.26–29.02)	27.81 (25.26–30.35)	0.88	0.92	0.86	0.54	0.61	0.56
Tertile 2 (11.700–13.985)	45/36/14	26.54 (24.87–28.21)	26.76 (25.17–28.36)	28.70 (24.48–32.93)	0.21	0.31	0.30			
Tertile 3 (>13.985)	36/44/11	28.21 (26.14–30.28)	28.80 (26.83–30.77)	28.86 (24.30–33.43)	0.77	0.66	0.63			
**Saturated fat (% of energy)**										
Tertile 1 (<8.595)	40/33/11	27.36 (25.63–29.09)	27.87 (25.90–29.85)	30.86 (25.74–35.99)	0.08	0.08	0.09	0.020	**0.015**	**0.016**
Tertile 2 (8.595–11.034)	47/42/15	26.62 (24.92–28.32)	28.64 (26.76–30.51)	28.44 (25.16–31.73)	0.30	0.17	0.16			
Tertile 3 (>11.034)	47/55/17	28.71 (27.05–30.36)	27.09 (25.77–28.41)	26.69 (24.05–29.33)	0.18	0.16	0.16			
**MUFA (% of energy)**										
Tertile 1 (<11.890)	43/37/12	27.70 (26.04–29.37)	28.17 (25.93–30.40)	27.58 (24.50–30.66)	0.95	0.99	0.91	0.82	0.94	0.99
Tertile 2 (11.890–14.571)	54/39/16	27.26 (25.64–28.88)	27.53 (26.13–28.93)	29.72 (26.56–32.89)	0.11	0.22	0.12			
Tertile 3 (>14.571)	37/54/15	27.88 (25.98–29.77)	27.72 (26.27–29.16)	27.55 (23.42–31.69)	0.85	0.95	0.99			
**PUFA (% of energy)**										
Tertile 1 (<8.426)	49/41/18	27.84 (25.94–29.74)	28.10 (26.08–30.11)	27.49 (23.97–31.00)	0.85	0.89	0.82	0.79	0.68	0.72
Tertile 2 (8.426–10.930)	49/43/15	26.38 (25.12–27.64)	28.06 (26.46–29.66)	29.42 (26.74–32.11)	0.033	0.037	0.034			
Tertile 3 (>10.930)	36/46/10	28.83 (26.93–30.74)	27.26 (25.83–28.69)	28.37 (23.58–33.17)	0.81	0.69	0.69			

^1^ *p*-value crude; ^2^ *p*-value adjusted for age and sex; ^3^ *p*-value adjusted for age, sex, and physical activity; *p*-values that are statistically significant are in bold.

## Supplementary Materials

The following are available online at https://www.mdpi.com/2075-4426/9/1/11/s1. Table S1: Associations of baseline characteristics, lifestyle and dietary habits with *FTO* (rs9939609), Table S2: Interaction between daily dietary intake and *FTO* (rs9939609) genotypes on body mass index (BMI) (kg/m^2^), Table S3: Interaction between daily dietary intake and *FTO* (rs1558902) genotypes on *body fat* (kg), Table S4: Interaction between daily dietary intake and *TCF7L2* (rs7903146) genotypes on body fat (kg), Table S5: Interaction between daily dietary intake and *FTO* (rs9939609) genotypes on body fat (kg), Table S6: Interaction between daily dietary intake and *FTO* (rs1558902) genotypes on muscle mass (kg), Table S7: Interaction between daily dietary intake and *TCF7L2* (rs7903146) genotypes on muscle mass (kg), Table S8: Interaction between daily dietary intake and *FTO* (rs9939609) genotypes on muscle mass (kg), Table S9: Interaction between physical activity and the three SNPs on body mass index (BMI) (kg/m^2^), body fat (kg), and muscle mass (kg).
