# Supplementary material for: The Interaction between Genetic Polymorphisms in FTO and TCF7L2 Genes and Dietary Intake with Regard to Body Mass and Composition: An Exploratory Study
Source: J Pers Med. 2019 Feb 5;9(1):11. doi: 10.3390/jpm9010011 (PMC6463113; doi:10.3390/jpm9010011)
Supplement: Supplementary file 1 [file jpm-09-00011-s001.pdf]

Table S1 Associations of baseline characteristics, lifestyle and dietary habits with ***FTO (rs9939609)***

		FTO (rs9939609)			
		TT	***TA***	***AA***	
		***N* = 91**	***N* = 158**	***N* = 58**	***P***
Characteristics and lifestyle factors					
**Gender, female**		54 (59.3)	99 (62.7)	40 (69.0)	0.49
**Age (years)**		38.61 ± 13.37	41.28 ± 14.53	37.99 ± 13.11	0.18
**Crowding index**		1.52 ± 0.88	1.54 ± 0.88	1.60 ± 0.76	0.86
**Levels of physical activity**	Low	33 (36.3)	75 (47.5)	29 (50.0)	0.40
	Moderate	34 (37.4)	47 (29.8)	18 (31.0)	
	High	24 (26.4)	36 (22.8)	11 (19.0)	
**Physical activity**	None	12 (13.2)	22 (13.9)	10 (17.2)	0.77
	Any	79 (86.8)	136 (86.1)	48 (82.8)	
**Cigarette smoker**	Never	46 (50.6)	75 (47.5)	27 (46.6)	0.44
	Current	35 (38.5)	70 (44.3)	29 (50.0)	
	Past	10 (11.0)	13 (8.2)	2 (3.5)	
**Narghileh smoker**	Never	49 (53.9)	94 (59.5)	34 (58.6)	0.93
	Current	32 (35.2)	50 (31.7)	18 (31.0)	
	Past	10 (11.0)	14 (8.9)	6 (10.3)	
**Alcohol drinker**	Never	63 (69.2)	113 (71.5)	46 (79.3)	0.15
	Current	23 (25.3)	37 (23.4)	6 (10.3)	
	Past	5 (5.5)	8 (5.1)	6 (10.3)	
Body mass and composition					
**Body mass index (BMI) (kg/m²)**		28.56 ± 5.56	27.07 ± 5.43	28.64 ± 5.99	0.06
**Body fat (kg)**		27.48 ± 11.56	24.91 ± 10.93	28.28 ± 11.99	0.08
**Muscle mass (kg)**		27.41 ± 6.66	26.22 ± 6.21	25.40 ± 6.27	0.15
Daily macronutrient intake					
**Total energy (Kcal)**		3739.09 ± 2043.47	3499.87 ± 2049.69	3619.59 ± 1968.32	0.67
**Carbohydrates (g)**		465.62 ± 266.87	432.46 ± 263.16	424.93 ± 208.40	0.53
**Percent Kcal from carbohydrates (%)**		50.87 ± 8.80	50.04 ± 7.77	48.96 ± 10.07	0.41
**Proteins (g)**		115.44 ± 65.10	111.94 ± 66.53	128.71 ± 113.47	0.37
**Percent Kcal from proteins (%)**		12.46 ± 2.65	13.06 ± 3.49	13.50 ± 5.17	0.21
**Sugar (g)**		143.44 ± 164.05	126.88 ± 96.44	127.81 ± 74.14	0.54
**Percent Kcal from sugar (%)**		14.80 ± 6.89	14.65 ± 6.12	15.04 ± 6.85	0.93
**Total Fat (g)**		150.35 ± 86.79	145.94 ± 86.74	158.84 ± 104.62	0.65
**Percent Kcal from total fat (%)**		37.92 ± 7.58	39.29 ± 7.50	39.79 ± 9.27	0.29
**Saturated fat (g)**		43.59 ± 27.77	41.08 ± 28.89	46.18 ± 31.84	0.50
**Percent Kcal from saturated fat (%)**		10.30 ± 2.68	10.27 ± 2.76	10.70 ± 2.86	0.58
**Monounsaturated fat (MUFA) (g)**		55.84 ± 33.26	53.73 ± 32.85	57.92 ± 40.19	0.71
**Percent kcal from MUFA (%)**		13.62 ± 4.02	13.85 ± 3.93	14.00 ± 4.39	0.84
**Polyunsaturated fat (PUFA) (g)**		38.13 ± 21.80	39.13 ± 24.54	41.63 ± 31.02	0.70
**Percent kcal from PUFA (%)**		9.31 ± 3.09	10.31 ± 3.95	10.28 ± 4.14	0.11
**Cholesterol (mg)**		408.11 ± 458.46	343.35 ± 273.28	440.40 ± 449.40	0.17

Data are presented as mean ± SD or *N* (%)

P values: chi-square, t-test or one way ANOVA as appropriate

Table S2. Interaction between daily dietary intake and ***FTO*** (***rs9939609***) genotypes on **BMI** (Kg/m^2)

	No. of subjects by genotype (*TT*/*TA*/*AA*)	*TT* Mean (95% CI) (*N* = 91)	*TA* Mean (95% CI) (*N* = 158)	*AA* Mean (95% CI) (*N* = 58)	*P*-trend[1]	*P*-trend[2]	*P*-trend[3]	*P*-interaction[1]	*P*-interaction[2]	*P*-interaction[3]
**Among ALL**	91/158/58	28.56 (27.40 – 29.71)	27.07 (26.21 – 27.92)	28.64 (27.07 – 30.22)	0.93	0.95	0.87	–	–	–
**Carbohydrates (% of energy)**										
Tertile 1 (<47.317)	30/48/25	28.87 (26.99 – 30.75)	27.30 (25.84 – 28.76)	29.42 (26.86 – 31.99)	0.70	0.96	0.96	0.46	0.60	0.59
Tertile 2 (47.317 – 53.984)	29/64/15	27.63 (25.64 – 29.62)	26.82 (25.43 – 28.20)	27.58 (24.20 – 30.96)	0.98	0.99	0.99			
Tertile 3 (>53.984)	32/46/18	29.10 (26.82 – 31.38)	27.18 (25.46 – 28.89)	28.44 (25.59 – 31.29)	0.71	0.66	0.66			
**Fat (% of energy)**										
Tertile 1 (<35.131)	32/43/17	28.83 (26.47 – 31.19)	26.84 (25.00 – 28.67)	29.07 (25.68 – 32.46)	0.90	0.94	0.94	0.49	0.57	0.46
Tertile 2 (35.131 – 41.333)	30/63/18	28.06 (26.28 – 29.84)	27.51 (26.14 – 28.88)	26.33 (24.15 – 28.51)	0.26	0.25	0.25			
Tertile 3 (>41.333)	29/52/23	28.77 (26.76 – 30.78)	26.72 (25.32 – 28.12)	30.13 (27.41 – 32.86)	0.39	0.43	0.43			
**Protein (% of energy)**										
Tertile 1 (<11.700)	39/58/23	29.27 (27.79 – 30.74)	26.73 (25.49 – 27.96)	28.53 (25.88 – 31.18)	0.57	0.40	0.40	0.94	0.77	0.76
Tertile 2 (11.700 – 13.985)	31/50/15	26.36 (24.50 – 28.21)	26.83 (25.21 – 28.46)	28.66 (25.45 – 31.87)	0.19	0.17	0.17			
Tertile 3 (>13.985)	21/50/20	30.48 (27.30 – 33.67)	27.70 (26.01 – 29.40)	28.76 (25.82 – 31.70)	0.38	0.23	0.23			
**Saturated fat (% of energy)**										
Tertile 1 (<8.595)	26/42/17	28.53 (26.82 – 30.24)	27.07 (25.12 – 29.02)	29.63 (26.29 – 32.97)	0.54	0.40	0.40	0.30	0.26	0.30
Tertile 2 (8.595 – 11.034)	29/65/10	28.22 (25.81 – 30.63)	27.00 (25.63 – 28.37)	30.74 (25.90 – 35.58)	0.25	0.31	0.31			
Tertile 3 (>11.034)	36/51/31	28.85 (26.87 – 30.83)	27.15 (25.85 – 28.46)	27.42 (25.47 – 29.38)	0.27	0.19	0.19			
**MUFA (% of energy)**										
Tertile 1 (<11.890)	30/41/21	28.99 (26.54 – 31.44)	26.48 (24.90 – 28.05)	29.00 (26.27 – 31.73)	1.00	0.93	0.93	0.75	0.87	0.81
Tertile 2 (11.890 – 14.571)	29/66/15	28.05 (26.10 – 30.00)	27.72 (26.40 – 29.05)	27.12 (23.64 – 30.60)	0.59	0.89	0.89			
Tertile 3 (>14.571)	32/51/22	28.61 (26.80 – 30.42)	26.69 (25.05 – 28.34)	29.33 (26.73 – 31.94)	0.64	0.95	0.95			
**PUFA (% of energy)**										
Tertile 1 (<8.426)	39/47/21	28.88 (26.09 – 30.94)	27.17 (25.19 – 29.14)	28.03 (25.11 – 30.94)	0.63	0.74	0.74	0.35	0.46	0.44
Tertile 2 (8.426 – 10.930)	29/61/18	28.18 (26.13 – 30.23)	26.91 (25.74 – 28.08)	28.39 (25.99 – 30.79)	0.89	0.44	0.44			
Tertile 3 (>10.930)	23/50/19	28.49 (26.61 – 30.38)	27.18 (25.71 – 28.64)	29.56 (26.34 – 32.78)	0.52	0.56	0.56			

[1]***p*-value crude**
[2]***p*-value adjusted for age and sex**
[3]***p*-value adjusted for age, sex and physical activity**

Table S3. Interaction between daily dietary intake and ***FTO*** (***rs1558902***) genotypes on **body fat** (kg)

	No. of subjects by genotype (*TT*/*TA*/*AA*)	*TT* Mean (95% CI) (*N* = 85)	*TA* Mean (95% CI) (*N* = 165)	*AA* Mean (95% CI) (*N* = 58)	*P*-trend[1]	*P*-trend[2]	*P*-trend[3]	*P*-interaction[1]	*P*-interaction[2]	*P*-interaction[3]
**Among ALL**	85/165/58	27.48 (24.95 – 30.00)	25.30 (23.59 – 27.00)	27.12 (24.01 – 30.24)	0.86	0.76	0.61	–	–	–
**Carbohydrates (% of energy)**										
Tertile 1 (<47.317)	25/53/26	26.06 (21.56 – 30.56)	25.19 (22.15 – 28.23)	27.50 (22.27 – 32.73)	0.66	0.86	0.83	0.16	0.26	0.24
Tertile 2 (47.317 – 53.984)	29/64/15	24.89 (20.90 – 28.89)	24.23 (21.48 – 26.99)	26.52 (20.02 – 33.02)	0.64	0.45	0.49			
Tertile 3 (>53.984)	31/48/17	31.03 (26.32 – 35.75)	26.84 (23.56 – 30.11)	27.08 (21.54 – 32.63)	0.27	0.32	0.26			
**Fat (% of energy)**										
Tertile 1 (<35.131)	31/45/16	29.23 (24.04 – 34.42)	24.22 (20.85 – 27.59)	29.90 (23.39 – 36.41)	0.86	0.89	0.98	0.61	0.63	0.49
Tertile 2 (35.131 – 41.333)	27/67/17	24.87 (20.81 – 28.94)	26.66 (23.90 – 29.42)	21.50 (17.28 – 25.72)	0.31	0.46	0.40			
Tertile 3 (>41.333)	27/53/25	28.06 (24.19 – 31.94)	24.49 (21.54 – 27.44)	29.17 (23.91 – 34.43)	0.72	0.84	0.84			
**Protein (% of energy)**										
Tertile 1 (<11.700)	39/57/25	29.44 (26.15 – 32.73)	25.54 (22.92 – 28.17)	25.48 (20.43 – 30.54)	0.14	0.09	0.07	0.29	0.20	0.18
Tertile 2 (11.700 – 13.985)	27/54/15	23.92 (20.17 – 27.67)	24.58 (21.47 – 27.69)	28.97 (22.87 – 35.06)	0.15	0.09	0.09			
Tertile 3 (>13.985)	19/54/18	28.49 (20.65 – 36.33)	25.76 (22.45 – 29.06)	27.87 (21.77 – 33.97)	0.89	0.52	0.39			
**Saturated fat (% of energy)**										
Tertile 1 (<8.595)	26/42/17	27.69 (24.00 – 31.38)	27.09 (23.17 – 31.01)	28.46 (21.68 – 35.24)	0.84	0.65	0.68	0.60	0.53	0.59
Tertile 2 (8.595 – 11.034)	28/65/11	26.96 (22.11 – 31.82)	25.12 (22.31 – 27.93)	28.28 (19.90 – 36.66)	0.75	0.98	0.87			
Tertile 3 (>11.034)	31/58/30	37.75 (22.93 – 32.58)	24.20 (21.68 – 26.72)	25.94 (21.81 – 30.08)	0.52	0.37	0.35			
**MUFA (% of energy)**										
Tertile 1 (<11.890)	29/44/19	30.61 (25.38 – 35.84)	23.63 (20.40 – 26.86)	29.84 (24.43 – 35.25)	0.83	0.94	0.80	0.54	0.58	0.48
Tertile 2 (11.890 – 14.571)	27/65/18	24.94 (20.52 – 29.36)	26.34 (23.75 – 28.92)	23.56 (17.69 – 29.42)	0.68	0.84	0.92			
Tertile 3 (>14.571)	29/56/21	26.70 (23.09 – 30.31)	25.40 (22.15 – 28.66)	27.73 (22.18 – 33.28)	0.76	0.98	0.87			
**PUFA (% of energy)**										
Tertile 1 (<8.426)	36/51/21	28.46 (23.97 – 32.94)	24.86 (21.27 – 28.45)	26.59 (20.92 – 32.26)	0.60	0.87	0.73	0.30	0.39	0.31
Tertile 2 (8.426 – 10.930)	26/66/16	27.75 (22.65 – 32.86)	24.83 (22.38 – 27.29)	26.79 (21.53 – 32.06)	0.78	0.42	0.20			
Tertile 3 (>10.930)	23/48/21	25.63 (22.33 – 28.92)	26.40 (23.27 – 29.52)	27.91 (21.97 – 33.84)	0.48	0.49	0.48			

[1]***p*-value crude**
[2]***p*-value adjusted for age and sex**
[3]***p*-value adjusted for age and sex and physical activity**

Table S4. Interaction between daily dietary intake and ***TCF7L2*** (***rs7903146***) genotypes on **body fat** (kg)

	No. of subjects by genotype (*CC/CT/TT*)	*CC* Mean (95% CI) (*N* = 134)	*CT* Mean (95% CI) (*N* = 130)	*TT* Mean (95% CI) (*N* = 43)	*P*- trend [1]	*P*- trend [2]	*P*- trend [3]	*P*- interaction[1]	*P*- interaction[2]	*P*- interaction[3]
**Among ALL**	134/130/43	26.27 (24.27 – 28.28)	26.15 (24.26 – 28.05)	26.32 (22.54 – 30.10)	0.98	0.94	0.95	–	–	–
**Carbohydrates (% of energy)**										
Tertile 1 (<47.317)	43/46/15	27.53 (23.83 – 31.22)	25.67 (22.52 – 28.82)	22.47 (15.77 – 29.17)	0.14	0.14	0.13	0.19	0.20	0.20
Tertile 2 (47.317 – 53.984)	44/49/15	22.81 (19.39 – 26.23)	26.20 (23.19 – 29.20)	25.57 (19.32 – 31.82)	0.40	0.31	0.26			
Tertile 3 (>53.984)	47/35/13	28.37 (25.02 – 31.73)	26.73 (22.57 – 30.90)	31.64 (24.00 – 39.28)	0.38	0.45	0.47			
**Fat (% of energy)**										
Tertile 1 (<35.131)	47/28/16	27.06 (23.24 – 30.87)	27.62 (22.19 – 33.05)	24.95 (20.03 – 29.87)	0.57	0.64	0.64	0.33	0.38	0.36
Tertile 2 (35.131 – 41.333)	46/51/14	22.98 (19.97 – 25.99)	26.03 (23.41 – 28.65)	31.34 (22.69 – 39.98)	**0.010**	0.031	0.023			
Tertile 3 (>41.333)	41/51/13	29.07 (25.52 – 32.62)	25.47 (22.48 – 28.46)	22.61 (15.64 – 29.58)	0.07	0.11	0.09			
**Protein (% of energy)**										
Tertile 1 (<11.700)	53/50/18	27.16 (23.90 – 30.42)	26.47 (23.68 – 29.27)	26.56 (22.09 – 31.04)	0.84	0.80	0.76	0.76	0.64	0.59
Tertile 2 (11.700 – 13.985)	45/36/14	25.50 (22.11 – 28.88)	23.68 (20.65 – 26.70)	26.98 (18.74 – 35.22)	0.66	0.62	0.61			
Tertile 3 (>13.985)	36/44/11	25.94 (21.76 – 30.12)	27.82 (23.81 – 31.82)	25.09 (14.90 – 35.28)	0.85	0.94	0.89			
**Saturated fat (% of energy)**										
Tertile 1 (<8.595)	40/33/11	26.54 (22.93 – 30.15)	26.82 (22.73 – 30.91)	33.15 (24.26 – 42.05)	0.10	0.10	0.11	0.030	**0.012**	**0.013**
Tertile 2 (8.595 – 11.034)	47/42/15	24.43 (21.16 – 27.71)	28.21 (24.58 – 31.83)	24.37 (16.91 – 31.82)	0.99	0.52	0.50			
Tertile 3 (>11.034)	47/55/17	27.89 (24.18 – 31.59)	24.18 (21.54 – 26.82)	23.62 (18.87 – 28.38)	0.17	0.10	0.10			
**MUFA (% of energy)**										
Tertile 1 (<11.890)	43/37/12	27.77 (24.05 – 31.49)	26.79 (22.41 – 31.17)	25.75 (19.51 – 31.99)	0.62	0.60	0.55	0.91	0.91	0.84
Tertile 2 (11.890 – 14.571)	54/39/16	24.64 (21.46 – 27.82)	25.18 (22.28 – 28.08)	29.14 (22.72 – 35.56)	0.15	0.11	0.07			
Tertile 3 (>14.571)	37/54/15	26.92 (23.11 – 30.72)	26.42 (23.51 – 29.33)	23.77 (15.82 – 31.72)	0.37	0.60	0.55			
**PUFA (% of energy)**										
Tertile 1 (<8.426)	49/41/18	26.93 (23.20 – 30.67)	26.18 (22.16 – 30.19)	25.43 (18.80 – 32.06)	0.67	0.81	0.76	0.97	0.71	0.74
Tertile 2 (8.426 – 10.930)	49/43/15	24.33 (21.43 – 27.24)	27.06 (23.64 – 30.49)	26.88 (20.59 – 33.17)	0.42	0.32	0.31			
Tertile 3 (>10.930)	36/46/10	28.02 (24.00 – 32.04)	25.28 (22.63 – 27.94)	27.09 (17.87 – 36.31)	0.81	0.49	0.50			

[1]*p*-value crude
[2]*p*-value adjusted for age and sex
[3]*p*-value adjusted for age and sex and physical activity

Table S5. Interaction between daily dietary intake and ***FTO*** (*rs9939609*) genotypes on **body fat** (kg)

	**No. of subjects by genotype (*TT/TA/AA*)**	***TT* Mean (95% CI) (*N* = 91)**	***TA* Mean (95% CI) (*N* = 158)**	***AA* Mean (95% CI) (*N* = 58)**	***P*-trend [1]**	***P*-trend [2]**	***P*-trend [3]**	***P*-interaction[1]**	***P*-interaction[2]**	***P*-interaction[3]**
**Among ALL**	91/158/58	27.47 (25.07 – 29.88)	24.91 (23.19 – 26.62)	28.28 (25.12 – 31.43)	0.67	0.86	0.97			
**Carbohydrates (% of energy)**										
Tertile 1 (<47.317)	30/48/25	26.08 (22.23 – 29.93)	24.78 (21.65 – 27.90)	28.89 (23.36 – 34.43)	0.36	0.71	0.71	0.14	0.21	0.20
Tertile 2 (47.317 – 53.984)	29/64/15	24.80 (20.68 – 28.92)	24.13 (21.42 – 26.83)	27.17 (20.62 – 33.71)	0.50	0.53	0.53			
Tertile 3 (>53.984)	32/46/18	31.21 (26.65 – 35.77)	26.12 (22.75 – 29.50)	28.34 (23.08 – 33.60)	0.41	0.37	0.37			
**Fat (% of energy)**										
Tertile 1 (<35.131)	32/43/17	29.42 (24.39 – 34.45)	24.17 (20.73 – 27.60)	29.05 (22.58 – 35.52)	0.92	0.77	0.77	0.32	0.32	0.24
Tertile 2 (35.131 – 41.333)	30/63/18	25.46 (21.61 – 29.30)	25.58 (22.71 – 28.45)	24.89 (20.20 – 29.58)	0.86	0.71	0.71			
Tertile 3 (>41.333)	29/52/23	27.41 (23.70 – 31.13)	24.70 (21.84 – 27.57)	30.36 (24.65 – 36.07)	0.33	0.40	0.40			
**Protein (% of energy)**										
Tertile 1 (<11.700)	39/58/23	29.49 (26.13 – 32.84)	24.81 (22.30 – 27.32)	28.05 (22.83 – 33.27)	0.60	0.30	0.30	0.52	0.32	0.31
Tertile 2 (11.700 – 13.985)	31/50/15	23.56 (20.06 – 27.06)	25.36 (22.10 – 28.61)	27.30 (20.86 – 33.74)	0.28	0.25	0.25			
Tertile 3 (>13.985)	21/50/20	29.52 (22.69 – 36.35)	24.57 (21.14 – 28.00)	29.27 (23.32 – 35.21)	0.95	0.57	0.57			
**Saturated fat (% of energy)**										
Tertile 1 (<8.595)	26/42/17	28.33 (24.67 – 31.99)	25.90 (21.97 – 29.83)	30.43 (23.97 – 36.89)	0.57	0.60	0.60	0.50	0.49	0.54
Tertile 2 (8.595 – 11.034)	29/65/10	27.11 (22.38 – 31.84)	24.44 (21.77 – 27.11)	32.38 (22.22 – 42.54)	0.22	0.21	0.21			
Tertile 3 (>11.034)	36/51/31	27.15 (22.86 – 31.44)	24.68 (21.94 – 27.42)	25.77 (21.86 – 29.68)	0.61	0.35	0.35			
**MUFA (% of energy)**										
Tertile 1 (<11.890)	30/41/21	30.37 (25.30 – 35.44)	23.65 (20.28 – 27.02)	29.23 (23.99 – 34.46)	0.74	0.61	0.61	0.36	0.42	0.37
Tertile 2 (11.890 – 14.571)	29/66/15	25.12 (20.83 – 29.41)	25.84 (23.26 – 28.41)	25.03 (18.40 – 31.67)	0.98	0.86	0.86			
Tertile 3 (>14.571)	32/51/22	26.90 (23.53 – 30.26)	24.72 (21.40 – 28.03)	29.58 (24.00 – 35.16)	0.39	0.79	0.79			
**PUFA (% of energy)**										
Tertile 1 (<8.426)	39/47/21	28.22 (23.96 – 32.48)	25.16 (21.46 – 28.85)	26.69 (20.90 – 32.47)	0.66	0.71	0.71	0.31	0.38	0.37
Tertile 2 (8.426 – 10.930)	29/61/18	27.36 (22.62 – 32.10)	24.03 (21.57 – 26.49)	29.46 (24.42 – 34.51)	0.51	0.68	0.68			
Tertile 3 (>10.930)	23/50/19	26.35 (23.35 – 29.36)	25.74 (22.64 – 28.85)	28.91 (22.56 – 35.27)	0.44	0.47	0.47			

[1]***p*-value crude**

[2]***p*-value adjusted for age and sex**

[3]***p*-value adjusted for age, sex and physical activity**

Table S6. Interaction between daily dietary intake and ***FTO*** (***rs1558902***) genotypes on **muscle mass** (kg)

	No. of subjects by genotype (*TT*/*TA*/*AA*)	*TT* Mean (95% CI) (*N* = 85)	*TA* Mean (95% CI) (*N* = 165)	*AA* Mean (95% CI) (*N* = 58)	*P*-trend[1]	*P*- trend [2]	*P*- trend [3]	*P*-interaction[1]	*P*-interaction[2]	*P*-interaction[3]
**Among ALL**	85/165/58	27.41 (25.96 – 28.85)	26.35 (25.40 – 27.30)	25.21 (23.57 – 26.85)	0.043	0.06	0.039	–	–	–
**Carbohydrates (% of energy)**										
Tertile 1 (<47.317)	25/53/26	29.48 (26.44 – 32.52)	27.79 (26.21 – 29.37)	26.08 (23.67 – 28.48)	0.05	0.14	0.13	0.55	0.97	1.00
Tertile 2 (47.317 – 53.984)	29/64/15	26.98 (24.50 – 29.46)	26.01 (24.33 – 27.69)	24.60 (21.04 – 28.16)	0.26	0.29	0.25			
Tertile 3 (>53.984)	31/48/17	26.14 (23.89 – 28.38)	25.20 (23.53 – 26.87)	24.42 (20.95 – 27.90)	0.35	0.26	0.22			
**Fat (% of energy)**										
Tertile 1 (<35.131)	31/45/16	27.39 (25.28 – 29.49)	26.87 (24.91 – 28.82)	25.62 (21.43 – 29.81)	0.38	0.69	0.63	0.83	0.96	0.80
Tertile 2 (35.131 – 41.333)	27/67/17	28.56 (25.37 – 31.75)	26.45 (24.91 – 27.99)	24.90 (21.77 – 28.03)	0.08	0.020	**0.016**			
Tertile 3 (>41.333)	27/53/25	26.27 (23.79 – 28.76)	25.78 (24.17 – 27.38)	25.16 (22.93 – 27.39)	0.49	0.61	0.60			
**Protein (% of energy)**										
Tertile 1 (<11.700)	39/57/25	27.52 (25.37 – 29.67)	24.80 (23.28 – 26.31)	24.51 (21.99 – 27.03)	0.06	0.13	0.08	0.86	0.72	0.75
Tertile 2 (11.700 – 13.985)	27/54/15	25.17 (22.95 – 27.39)	26.11 (24.40 – 27.82)	25.83 (21.97 – 29.68)	0.74	0.92	0.92			
Tertile 3 (>13.985)	19/54/18	30.36 (26.79 – 33.92)	28.22 (26.52 – 29.92)	25.67 (22.63 – 28.71)	0.029	0.07	0.05			
**Saturated fat (% of energy)**										
Tertile 1 (<8.595)	26/42/17	28.10 (25.17 – 31.04)	26.36 (24.53 – 28.19)	26.42 (22.32 – 30.51)	0.43	0.59	0.62	0.60	0.39	0.44
Tertile 2 (8.595 – 11.034)	28/65/11	25.36 (23.25 – 27.48)	25.76 (24.25 – 27.26)	22.86 (20.33 – 25.40)	0.22	0.37	0.25			
Tertile 3 (>11.034)	31/58/30	28.66 (26.09 – 31.24)	27.00 (25.28 – 28.73)	25.39 (23.21 – 27.56)	0.05	0.039	0.037			
**MUFA (% of energy)**										
Tertile 1 (<11.890)	29/44/19	27.82 (25.47 – 30.17)	25.98 (24.09 – 27.86)	24.65 (21.01 – 28.28)	0.10	0.26	0.20	0.65	0.65	0.55
Tertile 2 (11.890 – 14.571)	27/65/18	27.63 (24.81 – 30.45)	27.12 (25.52 – 28.71)	26.27 (23.33 – 29.21)	0.50	0.28	0.35			
Tertile 3 (>14.571)	29/56/21	26.79 (24.12 – 29.45)	25.75 (24.17 – 27.33)	24.81 (22.35 – 27.26)	0.26	0.38	0.30			
**PUFA (% of energy)**										
Tertile 1 (<8.426)	36/51/21	27.63 (25.56 – 29.70)	26.55 (24.67 – 28.43)	25.64 (22.23 – 29.06)	0.28	0.20	0.17	0.48	0.25	0.20
Tertile 2 (8.426 – 10.930)	26/66/16	29.38 (26.20 – 32.55)	26.95 (25.38 – 28.52)	24.80 (22.37 – 27.23)	0.029	0.040	0.017			
Tertile 3 (>10.930)	23/48/21	24.83 (22.44 – 27.21)	25.31 (23.76 – 26.85)	25.09 (22.24 – 27.94)	0.94	0.69	0.59			

[1]***p*-value crude**

[2]***p*-value adjusted for age and sex**

[3]***p*-value adjusted for age and sex and physical activity**

Table S7. Interaction between daily dietary intake and ***TCF7L2*** (***rs7903146***) genotypes on **muscle mass** (kg)

	**No. of subjects by genotype (*CC/CT/TT*)**	***CC* Mean (95% CI) (*N* = 134)**	***CT* Mean (95% CI) (*N* = 130)**	***TT* Mean (95% CI) (*N* = 43)**	***P*-trend[1]**	***P*-trend[2]**	***P*-trend[3]**	***P*-interaction[1]**	***P*-interaction[2]**	***P*-interaction[3]**
**Among ALL**	134/130/43	26.32 (25.27 – 27.37)	26.17 (25.07 – 27.27)	27.25 (25.07 – 29.43)	0.41	0.59	0.60	–	–	–
**Carbohydrates (% of energy)**										
Tertile 1 (<47.317)	43/46/15	29.03 (27.12 – 30.94)	26.67 (24.84 – 28.49)	27.54 (23.94 – 31.14)	0.43	0.18	0.17	0.23	0.05	0.05
Tertile 2 (47.317 – 53.984)	44/49/15	25.26 (26.56 – 26.97)	26.01 (24.12 – 27.90)	28.69 (23.77 – 33.61)	0.08	0.21	0.18			
Tertile 3 (>53.984)	47/35/13	24.84 (23.12 – 26.56)	25.75 (23.58 – 27.93)	25.26 (22.10 – 28.43)	0.82	0.41	0.42			
**Fat (% of energy)**										
Tertile 1 (<35.131)	47/28/16	25.72 (23.95 – 27.49)	27.55 (24.74 – 30.35)	28.13 (25.03 – 31.23)	0.20	0.59	0.59	0.19	0.08	0.07
Tertile 2 (35.131 – 41.333)	46/51/14	27.06 (25.06 – 29.06)	26.24 (24.49 – 27.99)	27.39 (22.19 – 32.60)	0.87	0.08	0.07			
Tertile 3 (>41.333)	41/51/13	26.19 (24.42 – 27.95)	25.35 (23.70 – 27.00)	26.02 (22.07 – 29.96)	0.93	0.24	0.20			
**Protein (% of energy)**										
Tertile 1 (<11.700)	53/50/18	26.36 (24.72 – 28.01)	24.75 (23.07 – 26.43)	25.79 (22.06 – 29.53)	0.74	0.95	0.99	0.29	0.78	0.73
Tertile 2 (11.700 – 13.985)	45/36/14	24.57 (22.95 – 26.19)	26.91 (24.64 – 29.17)	26.08 (22.56 – 29.60)	0.41	0.99	0.96			
Tertile 3 (>13.985)	36/44/11	28.45 (26.18 – 30.72)	27.18 (25.26 – 29.10)	31.13 (26.64 – 35.61)	0.24	0.33	0.32			
**Saturated fat (% of energy)**										
Tertile 1 (<8.595)	40/33/11	25.86 (23.90 – 27.81)	27.85 (25.36 – 30.35)	26.89 (21.84 – 31.94)	0.65	0.21	0.20	0.06	0.029	0.032
Tertile 2 (8.595 – 11.034)	47/42/15	24.64 (23.27 – 26.01)	24.87 (23.06 – 26.68)	28.89 (24.74 – 33.05)	**0.011**	0.13	0.11			
Tertile 3 (>11.034)	47/55/17	28.40 (26.37 – 30.44)	26.16 (24.50 – 27.81)	26.04 (22.66 – 29.41)	0.20	0.20	0.21			
**MUFA (% of energy)**										
Tertile 1 (<11.890)	43/37/12	25.73 (23.88 – 27.57)	27.21 (24.90 – 29.53)	25.42 (20.86 – 29.97)	0.89	0.81	0.84	0.86	0.38	0.42
Tertile 2 (11.890 – 14.571)	54/39/16	27.06 (25.32 – 28.79)	26.04 (24.14 – 27.94)	29.19 (25.06 – 33.33)	0.24	0.37	0.22			
Tertile 3 (>14.571)	37/54/15	25.95 (23.93 – 27.96)	25.56 (23.86 – 27.26)	26.65 (23.22 – 30.07)	0.71	0.69	0.65			
**PUFA (% of energy)**										
Tertile 1 (<8.426)	49/41/18	26.57 (24.67 – 28.47)	26.93 (24.70 – 29.17)	26.72 (23.72 – 29.72)	0.94	0.36	0.35	0.40	0.94	0.97
Tertile 2 (8.426 – 10.930)	49/43/15	26.37 (24.63 – 28.11)	26.70 (24.73 – 28.66)	30.77 (26.34 – 35.20)	0.023	0.027	0.026			
Tertile 3 (>10.930)	36/46/10	25.92 (23.98 – 27.86)	25.01 (23.38 –26.63)	22.92 (19.42 – 26.42)	0.13	0.41	0.46			

[1]*p*-value crude
[2]*p*-value adjusted for age and sex
[3]*p*-value adjusted for age and sex and physical activity

Table S8. Interaction between daily dietary intake and ***FTO*** (***rs9939609***) genotypes on **muscle mass** (kg)

	No. of subjects by genotype (*TT*/*TA*/*AA*)	*TT* Mean (95% CI) (*N* = 91)	*TA* Mean (95% CI) (*N* = 158)	*AA* Mean (95% CI) (*N* = 58)	*P*-trend[1]	*P*-trend[2]	*P*-trend[3]	*P*-interaction[1]	*P*-interaction[2]	*P*-interaction[3]
**Among ALL**	91/158/58	27.41 (26.02 – 28.80)	26.22 (25.24 – 27.20)	25.40 (23.75 – 27.05)	0.06	0.11	0.07	–	–	–
**Carbohydrates (% of energy)**										
Tertile 1 (<47.317)	30/48/25	29.14 (26.37 – 31.92)	27.39 (25.76 – 29.02)	26.79 (24.31 – 29.28)	0.17	0.38	0.38	0.59	0.88	0.89
Tertile 2 (47.317 – 53.984)	29/64/15	27.89 (25.44 – 30.34)	25.60 (23.93 – 27.27)	24.60 (21.10 – 28.10)	0.12	0.036	0.036			
Tertile 3 (>53.984)	32/46/18	25.35 (23.30 – 27.40)	25.86 (24.04 – 27.67)	24.14 (20.89 – 27.39)	0.50	0.63	0.63			
**Fat (% of energy)**										
Tertile 1 (<35.131)	32/43/17	27.17 (25.09 – 29.26)	27.03 (25.00 – 29.06)	25.65 (21.72 – 29.58)	0.44	0.83	0.83	0.84	0.99	0.88
Tertile 2 (35.131 – 41.333)	30/63/18	28.55 (25.66 – 31.44)	26.42 (24.80 – 28.04)	24.75 (21.83 – 27.67)	0.06	**0.014**	**0.014**			
Tertile 3 (>41.333)	29/52/23	26.49 (24.04 – 28.94)	25.30 (23.74 – 26.86)	25.73 (23.27 – 28.19)	0.64	0.75	0.75			
**Protein (% of energy)**										
Tertile 1 (<11.700)	39/58/23	27.00 (24.90 – 29.10)	25.22 (23.65 – 26.80)	24.11 (21.49 – 26.74)	0.08	0.27	0.27	0.94	0.49	0.50
Tertile 2 (11.700 – 13.985)	31/50/15	26.27 (24.08 – 28.46)	25.50 (23.76 – 27.25)	25.83 (22.04 – 29.62)	0.82	0.71	0.71			
Tertile 3 (>13.985)	21/50/20	29.86 (26.40 – 33.32)	28.09 (26.32 – 29.86)	26.56 (23.70 – 29.42)	0.11	0.07	0.07			
**Saturated fat (% of energy)**										
Tertile 1 (<8.595)	26/42/17	28.25 (25.40 – 31.11)	26.60 (24.74 – 28.46)	25.59 (21.48 – 29.69)	0.21	0.90	0.90	0.73	0.32	0.36
Tertile 2 (8.595 – 11.034)	29/65/10	25.37 (23.36 – 27.39)	25.25 (23.74 – 26.76)	25.88 (22.31 – 29.45)	0.81	0.64	0.64			
Tertile 3 (>11.034)	36/51/31	28.44 (26.03 – 30.86)	27.14 (25.31 – 26.97)	25.15 (23.03 – 27.26)	0.041	0.035	0.035			
**MUFA (% of energy)**										
Tertile 1 (<11.890)	30/41/21	27.55 (25.23 – 29.88)	26.02 (24.02 – 28.01)	24.98 (21.68 – 28.29)	0.17	0.13	0.13	0.81	0.56	0.52
Tertile 2 (11.890 – 14.571)	29/66/15	27.67 (24.94 – 30.39)	27.04 (25.48 – 28.59)	26.31 (22.96 – 29.66)	0.51	0.75	0.75			
Tertile 3 (>14.571)	32/51/22	27.04 (24.60 – 29.49)	25.32 (23.65 – 26.99)	25.19 (22.70 – 27.67)	0.28	0.37	0.37			
**PUFA (% of energy)**										
Tertile 1 (<8.426)	39/47/21	27.62 (25.57 – 29.67)	26.40 (24.45 – 28.35)	25.73 (22.32 – 29.14)	0.30	0.24	0.24	0.66	0.40	0.38
Tertile 2 (8.426 – 10.930)	29/61/18	28.43 (25.56 – 31.31)	27.29 (25.62 – 28.96)	24.99 (22.49 – 27.48)	0.22	0.08	0.08			
Tertile 3 (>10.930)	23/50/19	25.76 (23.17 – 28.34)	24.74 (23.28 – 26.20)	25.43 (22.46 – 28.41)	0.85	0.95	0.95			

[1]*p*-value crude
[2]*p*-value adjusted for age and sex
[3]*p*-value adjusted for age, sex and physical activity

Table S9. Interaction between **physical activity** and the three SNPs on **BMI** (kg/m^2), **body fat** (kg), and **muscle mass** (kg)

	Mean (95% CI)	Mean (95% CI)	Mean (95% CI)	*P*-trend[1]	*P*-trend[2]	*P*-interaction[1]	*P*-interaction[2]
FTO **(*rs1558902*)**	***TT***	***TA***	***AA***				
		**BMI** (kg/m^2)					
Low level of exercise	29.29 (27.06 – 31.52)	28.69 (27.27 – 30.11)	27.82 (25.72 – 29.93)	0.33	0.29	0.47	0.36
Moderate level of exercise	28.94 (27.33 – 30.54)	27.05 (25.78 – 28.32)	28.29 (25.79 – 30.79)	0.65	0.87		
High level of exercise	26.73 (24.17 – 29.29)	25.26 (23.76 – 26.76)	27.82 (21.99 – 33.65)	0.61	0.38		
		**Body fat** (kg)					
Low level of exercise	28.80 (24.51 – 33.10)	28.01 (25.21 – 30.82)	27.46 (23.44 – 31.49)	0.65	0.49	0.94	0.72
Moderate level of exercise	27.42 (23.96 – 30.88)	24.99 (22.34 – 27.64)	26.89 (21.73 – 32.05)	0.86	0.84		
High level of exercise	25.52 (19.64 – 31.40)	20.60 (17.41 – 23.80)	26.55 (14.92 – 38.19)	0.82	0.63		
		**Muscle mass** (kg)					
Low level of exercise	27.39 (25.17 – 29.62)	26.68 (25.23 – 28.13)	24.52 (22.03 – 27.01)	0.07	0.07	0.48	0.79
Moderate level of exercise	28.26 (25.54 – 30.99)	26.11 (24.39 – 27.83)	26.26 (22.96 – 29.56)	0.32	0.69		
High level of exercise	26.42(23.39 – 29.45)	26.06 (24.06 – 28.05)	25.47 (22.14 – 28.81)	0.68	0.43		
TCF7L2 **(*rs7903146*)**	***CC***	***CT***	***TT***				
		**Body mass index (BMI)** (kg/m^2)					
Low level of exercise	28.87 (27.08 – 30.67)	28.54 (27.17 – 29.91)	28.46 (25.05 – 31.86)	0.80	0.84	0.38	0.47
Moderate level of exercise	27.17 (25.88 – 28.46)	28.51 (26.83 – 30.20)	28.07 (25.56 – 30.57)	0.52	0.65		
High level of exercise	26.17 (24.15 – 28.20)	25.08 (23.18 – 26.97)	28.61 (23.54 – 33.68)	0.23	0.33		
		**Body fat** (kg)					
Low level of exercise	26.94 (25.49 – 32.38)	27.64 (24.91 – 30.38)	27.42 (21.11 – 33.73)	0.64	0.67	0.69	0.64
Moderate level of exercise	24.78 (21.99 – 27.57)	28.07 (24.99 – 31.16)	24.86 (18.94 – 30.79)	0.98	0.99		
High level of exercise	24.42 (19.78 – 29.07)	20.20 (16.14 – 24.27)	26.38 (16.44 – 36.32)	0.65	0.58		
		**Muscle mass** (kg)					
Low level of exercise	26.51 (24.75 – 28.27)	26.54 (24.92 – 28.16)	25.48 (22.56 – 28.40)	0.56	0.33	0.16	0.24
Moderate level of exercise	26.90 (25.25 – 28.56)	25.35 (23.33 – 27.38)	28.64 (23.18 – 34.09)	0.38	0.40		
High level of exercise	25.16 (22.86 – 27.45)	26.28 (23.91 – 28.64)	28.38 (24.91 – 31.85)	0.14	0.19		
FTO (rs9939609)	***TT***	***TA***	***AA***				
		**Body mass index (BMI)** (kg/m^2)					
Low level of exercise	29.53 (27.21 – 31.85)	28.37 (27.00 – 29.73)	28.40 (26.16 – 30.63)	0.46	0.46	0.48	0.48
Moderate level of exercise	28.76 (27.37 – 30.14)	26.51 (25.13 – 27.90)	29.22 (26.89 – 31.56)	0.72	0.72		
High level of exercise	26.93 (24.45 – 29.41)	25.09 (23.56 – 26.63)	28.34 (22.74 – 33.93)	0.49	0.41		
		**Body fat** (kg)					
Low level of exercise	28.98 (24.40 – 33.55)	27.60 (24.96 – 30.24)	28.37 (23.99 – 31.76)	0.84	0.82	0.94	0.89
Moderate level of exercise	27.11 (24.12 – 30.09)	24.01 (21.09 – 26.93)	29.00 (24.23 – 33.76)	0.49	0.67		
High level of exercise	25.93 (20.25 – 31.62)	20.47 (17.25 – 23.69)	26.85 (15.45 – 38.25)	0.83	0.93		
		**Muscle mass** (kg)					
Low level of exercise	27.60 (25.32 – 29.89)	26.24 (24.82 – 27.66)	25.39 (22.77 – 28.02)	0.17	0.04	0.88	0.78
Moderate level of exercise	27.98 (25.60 – 30.37)	25.94 (24.05 – 27.82)	26.40 (23.35 – 29.45)	0.40	0.94		
High level of exercise	26.33 (23.44 – 29.23)	26.54 (24.47 – 28.62)	23.79 (20.63 – 26.96)	0.26	0.48		

[1]*p*-value crude [2]*p*-value adjusted for age and sex
